# The Diabetic-Thyroid Link: A Cross-Sectional Study of Thyroid Dysfunction Prevalence in a Diabetic Population

**DOI:** 10.7759/cureus.95446

**Published:** 2025-10-26

**Authors:** Parth Jani, Niyati N Pandya, Kuldeepkumar Viramgama, Shabir Badi, Dipak Dangar, Pankaj Akholkar

**Affiliations:** 1 Internal Medicine, All India Institute of Medical Sciences, Rajkot, Rajkot, IND; 2 Anaesthesiology, All India Institute of Medical Sciences, Rajkot, Rajkot, IND; 3 General Medicine, Government Medical College, Bhavnagar, IND

**Keywords:** diabetes mellitus, insulin therapy, prevalence, subclinical hypothyroidism, thyroid dysfunction

## Abstract

Objectives: This study aimed to assess the prevalence and patterns of thyroid dysfunction in patients with diabetes mellitus (DM) and analyze its association with demographic and clinical factors.

Materials and methods: A cross-sectional observational study was conducted on 280 diabetic patients without pre-existing thyroid disease at a tertiary care hospital. Participants underwent clinical evaluation and biochemical assessment of fasting blood sugar, postprandial blood sugar, and thyroid function tests (thyroid-stimulating hormone (TSH), free thyroxine (FT3), free triiodothyronine (FT4)) via chemiluminescent immunoassays. Thyroid dysfunction was classified based on standard definitions.

Results: The prevalence of thyroid dysfunction was 30.35%. Subclinical hypothyroidism (SCH) was the most common abnormality (13.22%), followed by overt hypothyroidism (8.21%). A statistically significant association was found between thyroid dysfunction and female gender (p = 0.0091) and insulin therapy (p<0.0001). No significant correlation was observed with age, duration of DM, family history of DM, or body mass index.

Conclusions: Thyroid dysfunction is highly prevalent in diabetic patients, with SCH being predominant. Female patients and those on insulin therapy are at higher risk. Routine screening for thyroid disorders is recommended in all patients with DM.

## Introduction

Diabetes mellitus (DM) represents a spectrum of chronic metabolic disorders characterized by hyperglycemia resulting from defects in insulin secretion, insulin action, or, frequently, a combination of both. The pathophysiological mechanisms, however, differ significantly between the two primary types. Type 1 DM (T1DM) is an autoimmune condition marked by the absolute deficiency of insulin due to the destruction of pancreatic beta-cells. In contrast, type 2 DM (T2DM) is defined by a dual pathology of progressive insulin resistance in peripheral tissues and a relative insulin deficiency due to beta-cell dysfunction. This chronic hyperglycemia imposes a substantial global health burden, leading to multi-organ complications, increased morbidity and mortality, and significant economic costs, often measured in disability-adjusted life years (DALYs). The situation is particularly acute in India, where the burden is escalating rapidly. World Health Organization estimates have risen from 31.7 million cases in 2000 to 50.8 million in 2010, with a projection of 87 million by 2030 [1]. Concurrently, the Indian Council of Medical Research (ICMR)'s 2011 findings revealed a significant burden of dysglycemia, with approximately 77.2 million individuals having prediabetes and 62.4 million with diabetes [2]. This rising prevalence of diabetes directly implies a parallel increase in the population at risk for its associated comorbidities, including endocrine disorders such as thyroid dysfunction, thereby necessitating targeted research into these interconnected health challenges.

Thyroid dysfunction, the second most common endocrine disorder after DM [3], demonstrates a significantly higher prevalence in individuals with diabetes compared to the non-diabetic population [3,4]. The association with T1DM is well-established and primarily driven by shared autoimmune mechanisms. While the link with T2DM is less clearly defined, potential contributing factors include an altered thyrotropin-releasing hormone (TRH) response, loss of the nocturnal thyroid-stimulating hormone (TSH) surge, and a low triiodothyronine (T3) state often associated with insulin resistance [3,4]. Importantly, the interplay is bidirectional. Hyperthyroidism is well-known to disrupt glucose homeostasis through multiple pathways: it increases gluconeogenesis and glycogenolysis, enhances intestinal glucose absorption, and can cause peripheral insulin resistance, thereby predisposing to hyperglycemia and worsening glycemic control in diabetic patients. This complex relationship underscores why the co-existence of both conditions necessitates integrated management. Furthermore, undiagnosed thyroid disease can impede glycemic control, exacerbate diabetes-related complications, and negatively impact overall patient outcomes [3,5-7]. Therefore, the recognition and management of thyroid disorders are integral to optimizing diabetes care.

The wide variability in reported prevalence of thyroid dysfunction in diabetic populations (ranging from 2.2% to 31%) [8,9] can be attributed to methodological differences across studies, including variations in diagnostic criteria for thyroid disease, the demographic composition of the study population (e.g., age, gender, iodine status), and whether screening included autoimmune markers. While subclinical hypothyroidism (SCH) is consistently reported as the most common abnormality [10,11], its clinical significance must be emphasized; it is not merely a biochemical diagnosis but a state associated with an increased risk of progression to overt hypothyroidism, adverse effects on lipid profiles, and an elevated cardiovascular risk [3,5-7].

Despite the recognized association and clinical significance, data on the prevalence and specific patterns of thyroid dysfunction within diabetic populations, particularly in specific regional healthcare settings like ours, remain limited. Our study focuses specifically on a hospital-based cohort of patients with T2DM, as this constitutes the overwhelming majority of the diabetes burden in India. Understanding the local epidemiology is crucial for developing context-appropriate screening protocols and informing resource allocation within our healthcare system.

The aim of this study is to assess the relationship between DM and thyroid dysfunction in patients admitted to Sir Takhatsingh General Hospital, Bhavnagar, India. Our specific objectives are the following: (1) to assess the prevalence of thyroid dysfunction in patients diagnosed with DM; and (2) to analyze the distribution of thyroid dysfunction across demographic (age, sex) and clinical factors (duration of DM, family history of thyroid disorders/DM, treatment regularity, body mass index (BMI)).

This hospital-based observational study will utilize patient records to address these objectives and contribute to the understanding of this comorbidity in our local context.

## Materials and methods

This hospital-based, cross-sectional observational study was conducted over a nine-month period at the outpatient department of general medicine at Sir Takhtasinhji General Hospital, Bhavnagar. The primary aim was to evaluate the prevalence and pattern of thyroid dysfunction in patients with a known diagnosis of DM.

Study population and sample size calculation

The study group comprised 280 adult patients (aged 18 years and above) diagnosed with DM. The sample size was determined to ensure that the results were statistically reliable and representative. Based on a review of existing literature, the anticipated prevalence of thyroid dysfunction in diabetic populations was estimated to be approximately 27% [12]. Using this proportion (p = 0.27) with a 95% confidence level (Z = 1.96) and a relative precision (d) of 5%, the minimum required sample size was calculated using the formula for cross-sectional studies: n = (Z² * p * (1-p)) / d². This calculation yielded a sample size of approximately 303. A final sample of 280 patients was enrolled during the study period. This achieved sample size provides a slightly revised precision of 5.2% for the primary prevalence objective and was deemed statistically adequate and practically achievable. This sample size provides a power of over 80% to detect a 15% difference in prevalence between key subgroups (e.g., males vs. females) with a significance level (alpha) of 0.05.

Inclusion and exclusion criteria

Patients were enrolled consecutively as they presented to the outpatient clinics. The inclusion criterion was a confirmed diagnosis of DM, based on either the American Diabetes Association/WHO criteria (elevated fasting plasma glucose of ≥126 mg/dL, two-hour postprandial glucose of ≥200 mg/dL, HbA1c of ≥6.5%, or random plasma glucose of ≥200 mg/dL with classic symptoms) or current use of anti-diabetic medication.

Several exclusion criteria were applied to minimize confounding factors. These included the following: a known history of any pre-existing thyroid disorder; chronic kidney failure or diabetic nephropathy (to exclude non-thyroidal illness syndrome); critical illnesses such as sepsis, acute myocardial infarction, or severe heart failure; admission to an intensive care unit (ICU) within the preceding 24 hours [13]; moderate-to-severe hepatic dysfunction, defined as alanine aminotransferase (ALT) or aspartate aminotransferase (AST) levels greater than five times the upper limit of normal (ULN) [14]; psychiatric illness; and pregnancy. Additionally, patients currently taking medications known to interfere with thyroid function tests (e.g., amiodarone, propranolol, corticosteroids, oral contraceptives) were excluded. Patients unwilling to provide informed consent were also not enrolled.

Study parameters and data collection

Each participant underwent a comprehensive assessment. Data collection involved a detailed history, clinical examination, and biochemical investigations. The demographic and clinical parameters recorded included age, gender, duration of diabetes, family history of diabetes, details of anti-diabetic treatment (including type of medication and adherence: it was assessed based on patient self-report, corroborated with prescription records), and associated comorbidities. A thorough general and systemic examination was performed. Anthropometric measurements were taken, with a BMI calculated as weight in kilograms divided by the square of height in meters (kg/m²). Participants were classified according to the WHO Asia-Pacific guidelines: underweight (BMI < 18.5), normal (18.5-22.9), overweight (23-24.9), and obese (≥25).

Biochemical evaluation

Biochemical investigations were central to the study. After an overnight fast of at least eight hours, venous blood samples were collected. Fasting blood sugar (FBS) and postprandial blood sugar (PPBS), measured two hours after a meal, were analyzed using the glucose oxidase (Trinder's) method. Serum levels of TSH, free thyroxine (FT4), and free triiodothyronine (FT3) were measured. The reference intervals, as established and validated by the Clinical Laboratory for the Chemiluminescent Immunoassays (CLIA) on a fully automated Mindray CL-900i analyzer platform, were as follows: FT3 of 1.7-3.7 pg/mL, FT4 of 0.7-1.48 ng/dL, and TSH of 0.39-4.9 mIU/L.

Definitions of thyroid dysfunction

Thyroid dysfunction was classified biochemically according to established criteria. Overt hyperthyroidism was defined as a markedly suppressed TSH level (<0.1 mIU/L) with an elevated FT4 (>1.48 ng/dL). Subclinical hyperthyroidism was characterized by a low or suppressed TSH (<0.4 mIU/L), with FT3 and FT4 levels within their normal reference ranges. Conversely, overt hypothyroidism was defined as a markedly elevated TSH (≥10 mIU/L) with a low FT4 (<0.7 ng/dL). SCH was identified by an elevated TSH level (>4.5 mIU/L), with both FT3 and FT4 levels within the normal range, consistent with standard clinical definitions [15].

Statistical analysis

Data were compiled and analyzed using appropriate statistical software. Descriptive statistics were presented as mean ± standard deviation (SD) for continuous variables and as frequencies and percentages for categorical variables. Inferential statistics were used to test associations. The chi-square test (or Fisher's exact test where expected cell counts were less than five) was employed to compare categorical variables (e.g., thyroid dysfunction vs. gender, treatment type). Student's t-test was used to compare means of continuous variables (e.g., age, BMI) between groups with normal and abnormal thyroid function. A p-value of less than 0.05 (p < 0.05) was considered statistically significant throughout the analysis.

Ethical considerations

The study protocol was reviewed and approved by the Institutional Ethics Committee (IEC) of Sir Takhtasinhji General Hospital, Bhavnagar. Written informed consent was obtained from every participant after a detailed explanation of the study procedures. Confidentiality was maintained by anonymizing all patient data.

## Results

**Figure 1:**
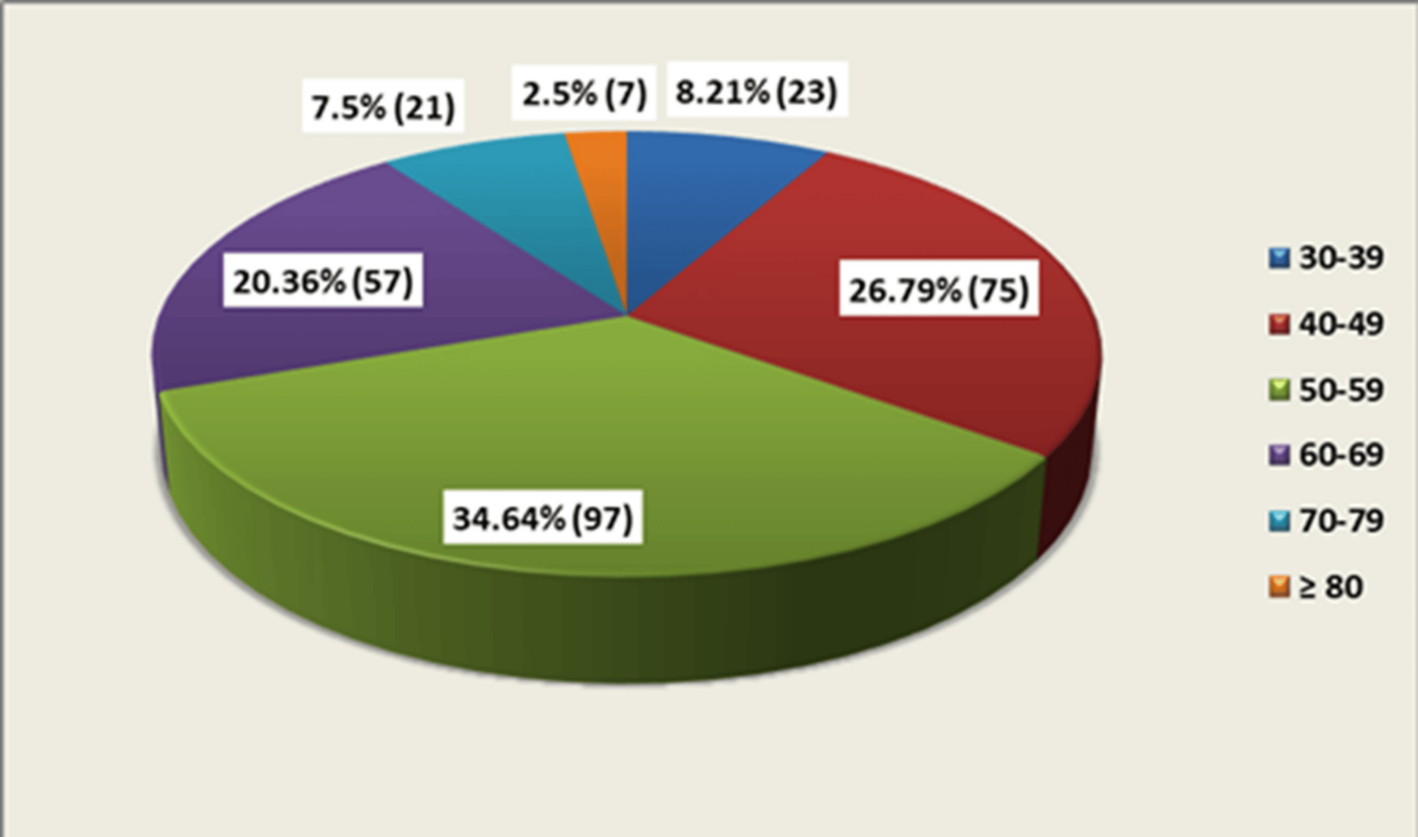
Age distribution of patients

**Figure 2:**
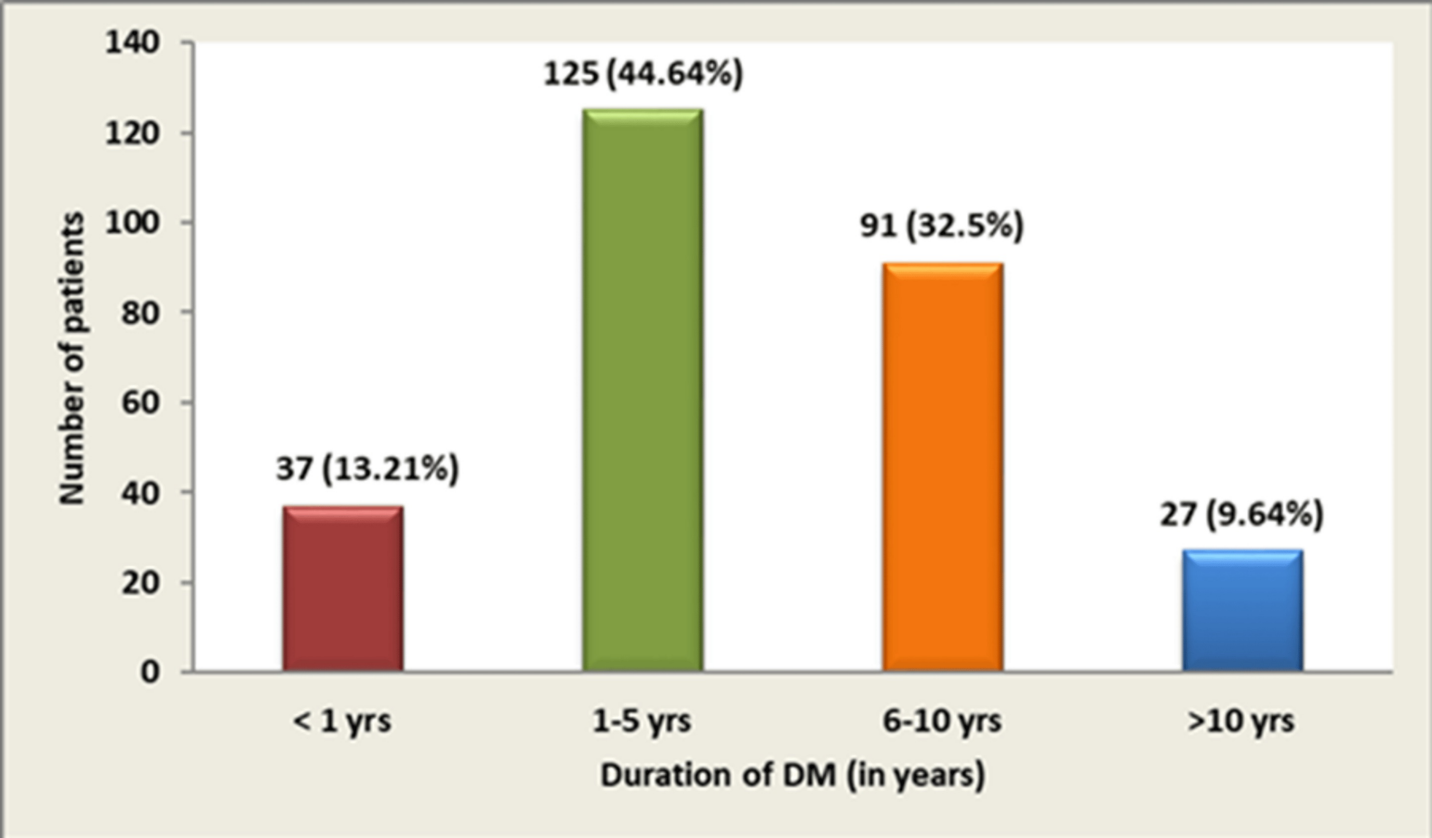
Duration of DM-wise distribution DM: diabetes mellitus

Participants aged 32-86 years (mean: 54.03 ± 10.93) were analyzed. Most individuals (172; 61.43%) belonged to the 40-59 year age group (Figure 1). Males accounted for 56.42% (158/280) of DM cases and females 43.57% (122/280), establishing a 1.29:1 male-female ratio. Among study participants, 162 (57.85%) had diabetes for ≤5 years, 91 (32.5%) for 6-10 years, and 27 (9.64%) for >10 years. The most frequent demographic was 41-60 year-olds with <5 years' duration (Figure 2).

**Figure 3:**
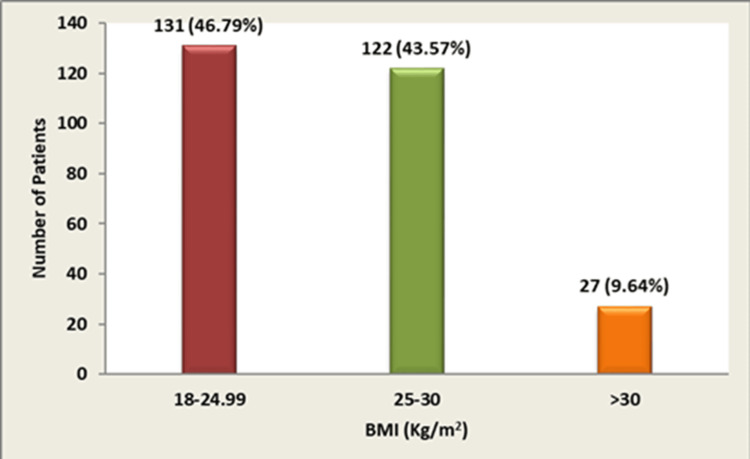
BMI-wise distribution

**Figure 4:**
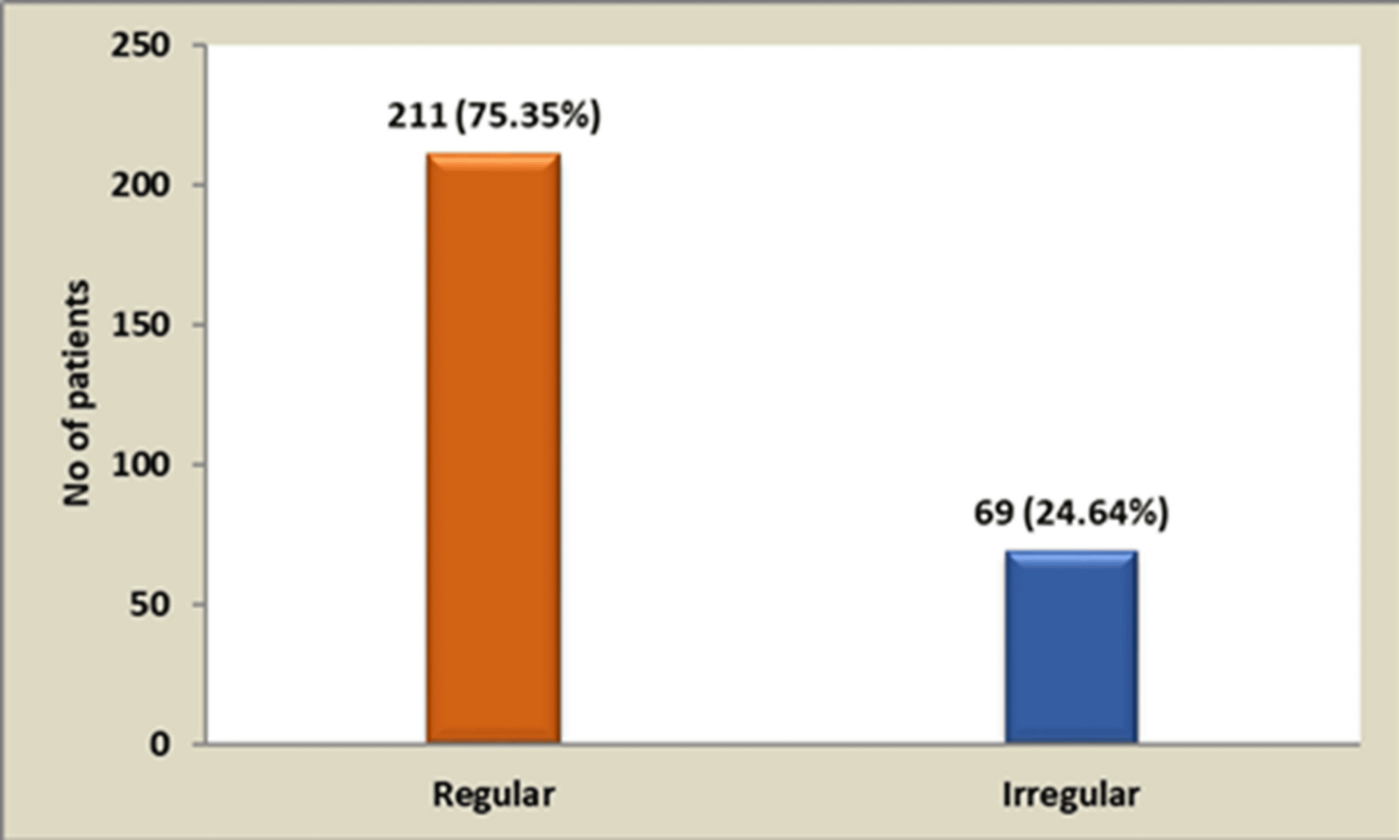
Treatment-wise distribution

Among study participants, 47.14% (n = 132) had a diabetic family history; the remaining 52.85 % (n = 148) reported no familial history. In this cohort, 53.21% (n = 149) exceeded normal BMI thresholds, whereas 46.79% (n = 131) fell within the normal range (Figure 3). Regular treatment adherence was observed in 211/280 participants (75.35%), contrasting with 69/280 (24.64%) exhibiting irregular adherence (Figure 4).

**Figure 5:**
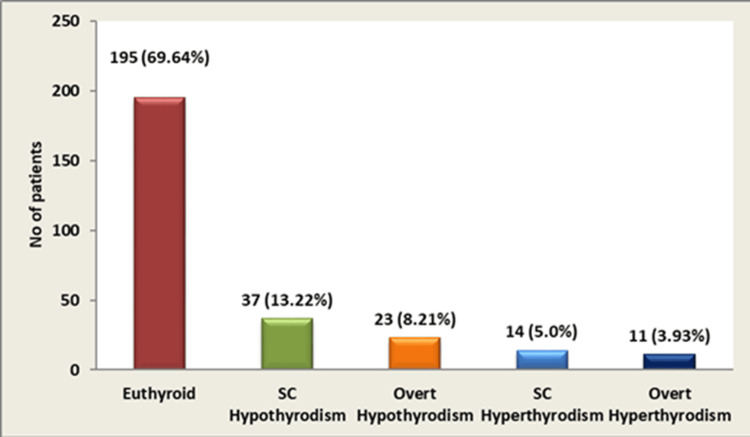
Distribution of thyroid function

Among study participants, 13.22% (n = 37) presented with SCH, 8.21% (n = 23) with overt hypothyroidism, 5.0% (n = 14) with subclinical hyperthyroidism, and 3.93% (n = 11) with overt hyperthyroidism. The majority (69.64%, n = 195) maintained normal thyroid function (Figure 5).

In Table 1, the correlation of thyroid profile with diabetic patients presents a comparative analysis of various parameters between patients with normal thyroid function and those with abnormal thyroid function. The analysis revealed that the mean age of patients with normal TSH (54.01 ± 10.88 years) was nearly identical to that of patients with abnormal TSH (54.10 ± 11.02 years), and this difference was not statistically significant (p = 0.9495, Student's t-test). Similarly, the duration of diabetes showed no significant correlation with thyroid status, with mean durations of 5.73 ± 4.5 years and 5.40 ± 3.7 years in the normal and abnormal TSH groups, respectively (p = 0.5583, Student's t-test).

**Table 1:** Correlation of thyroid profile with diabetic patients Comparison of various parameters between diabetic patients with normal and abnormal thyroid function. Data are presented as mean ± standard deviation for continuous variables and as number (percentage) for categorical variables. Units: Age (years), Duration of DM (years), BMI (kg/m²). A p-value < 0.05 was considered statistically significant. Statistical tests used: Student's t-test for age, duration of DM, and BMI; chi-square test for gender, family history, and treatment.

Variables	Normal TSH (n=195)	Abnormal TSH (n=85)	P value
Age (year)	54.01 ± 10.88	54.10 ± 11.02	0.950
Gender (Male:Female)	120 (61.5%):75 (38.4%)	38 (44.7%):47 (55.2%)	0.009
Duration of DM (yrs)	5.73 ± 4.5	5.40 ± 3.7	0.558
Family History of DM (Yes:No)	97 (49.7%):98 (50.2%)	35 (41.1%):50 (58.8%)	0.187
Treatment (Regular:Irregular)	169 (86.6%):26 (13.3%)	42 (49.4%):43 (50.8%)	<0.001
BMI (kg/m^2^)	25.69 ± 2.8	26.01 ± 3.01	0.399

A highly significant association was found with gender (p = 0.0091, chi-square test). While males constituted a larger proportion of the normal TSH group (61.5%), females represented a higher percentage (55.2%) within the group with thyroid dysfunction, underscoring a female predisposition. Furthermore, a very strong association was observed with treatment patterns (p<0.0001; chi-square test). An overwhelming majority (86.6%) of patients with normal thyroid function were on regular treatment, whereas the group with thyroid dysfunction had a nearly equal split, with 50.8% reporting irregular treatment adherence. This suggests that insulin therapy, often indicative of more advanced or difficult-to-control diabetes, is a significant correlate of thyroid dysfunction. Other factors, including family history of diabetes and BMI, showed no statistically significant association with thyroid status in this cohort (p = 0.1866 and p = 0.3998, respectively).

## Discussion

The present cross-sectional analysis determined the prevalence and patterns of thyroid dysfunction in 280 patients with DM without pre-existing thyroid disorders. Our key findings reveal a 30.35% prevalence of thyroid dysfunction, predominantly SCH (13.22% of total, 43.52% of abnormalities), followed by overt hypothyroidism (8.21%). Significant correlations were established between thyroid dysfunction and female gender, as well as insulin therapy. No significant correlations were found with age, duration of DM, family history of DM, or BMI.

Prevalence of thyroid dysfunction

We observed thyroid dysfunction in 30.35% of the diabetic cohort, a finding consistent with global reports, including Diez et al. (Spain, 32.4%) [16], Pasupthi et al. (45%) [17], Vinu et al. (28.75%) [18], and Agrawal et al. (27.8%) [19]. This consistently high prevalence across diverse populations underscores thyroid dysfunction as a major comorbidity in DM. While the strong association between autoimmune thyroiditis and T1DM is well-established, our data indicate a significant burden in T2DM as well. Potential mechanistic links include shared autoimmune pathogenesis, hyperglycemia-induced alterations in the hypothalamic-pituitary-thyroid axis (e.g., reduced deiodinase activity and altered T3/reverse triiodothyronine (rT3) levels), and impaired TSH response to TRH in poorly controlled diabetes.

Pattern of thyroid dysfunction

SCH emerged as the predominant abnormality, affecting 13.22% of the total cohort and constituting 43.52% of all thyroid dysfunctions. This aligns with reports by Chander et al. (14.7% SCH) [20] and Raguvanshi et al. (15% SCH) [12]. Overt hypothyroidism was present in 8.21% of patients, comparable to rates reported by Chander et al. (10%) [20] and Raguvanshi et al. (10%) [12]. Hyperthyroidism was less common, with subclinical hyperthyroidism at 5% and overt hyperthyroidism at 3.93%, similar to Demitrost et al. (subclinical 2%, clinical 1.5%) [10] and Chander et al. (subclinical 2%, clinical 0.6%) [20]. This pattern highlights SCH as a critical entity in diabetic patients, warranting vigilant monitoring due to its potential progression to overt disease and its established association with increased cardiovascular risk and dyslipidemia - comorbidities already elevated in the diabetic population.

Association with gender and insulin treatment

A significant gender disparity was evident, with females exhibiting a higher prevalence of thyroid dysfunction (55.29% of abnormalities) compared to males (44.70%) (p = 0.0091). This mirrors the well-established female predominance of thyroid disorders in the general population, as also noted by Demitrost et al. [10] in diabetics, and likely reflects underlying hormonal and autoimmune factors. Furthermore, a highly significant association (p < 0.0001) was found between abnormal thyroid function and insulin therapy. This association likely signifies greater diabetes severity, longer duration, or poorer beta-cell function in insulin-requiring patients, rather than a direct effect of insulin on thyroid function. These patients may constitute a distinct clinical subset exhibiting more severe metabolic derangement impacting the thyroid axis or a higher burden of autoimmunity.

Lack of association with other parameters

Our analysis revealed no significant correlations between thyroid dysfunction and several other parameters within this diabetic cohort. The mean age of participants (54.03 ± 10.93 years) was consistent with other Indian studies (Sarode et al. [21]: 55.09 ± 10.99; Chander et al. [20]: 50.69 ± 11.48; Reddy et al. [22]: 55.98 ± 11.19), but unlike trends in the general population, thyroid dysfunction prevalence did not increase with age in our diabetic sample, aligning with Chander et al. [20] Most patients (57.85%) had diabetes for ≤5 years. Despite the theoretical impact of hyperglycemia on thyroid hormone metabolism (e.g., T4 to T3 conversion), we found no significant link between DM duration and thyroid dysfunction, consistent with Chander et al. [20]. Nearly half the cohort (47.14%) reported a family history of DM, similar to rates noted by Tattersal et al. [23] and Vishwanathan et al. [24], yet this was not predictive of thyroid dysfunction. Over half the patients (53.21%) were overweight or obese (mean BMI: 25.79 ± 2.91 kg/m²). Although Reddy et al. [22] observed a positive correlation between BMI elevation and thyroid dysfunction prevalence in the diabetic population, our study found no significant correlation.

New findings and advantages

This study provides contemporary data on the high prevalence (30.35%) and pattern (SCH predominance) of thyroid dysfunction in a well-defined cohort of Indian diabetic patients without prior thyroid diagnosis, using standardized chemiluminescence assays. It reinforces the strong female predisposition within the diabetic population. Furthermore, the strong statistical association (p < 0.0001) between thyroid dysfunction and insulin therapy likely identifies a patient subgroup with more advanced or difficult-to-control diabetes that warrants closer thyroid monitoring.

Limitations

Cross-sectional design: The study establishes the association but not causation and cannot determine if DM predisposes to thyroid dysfunction, vice versa, or if shared factors cause both.

Single-center study: The study was conducted at Sir Takhtasinhji General Hospital, Bhavnagar, potentially limiting generalizability to other regions or healthcare settings.

Lack of autoimmune markers: The study did not include measurement of thyroid peroxidase antibodies (TPO-Ab) or thyroglobulin antibodies (Tg-Ab). Autoimmunity is a major cause of thyroid dysfunction, especially in T1DM and SCH, and its contribution here remains unexplored.

Glycemic control: Glycosylated hemoglobin (HbA1c), widely recognized as the reference standard for long-term glycemic control evaluation, was omitted. The impact of glycemic control dynamics (severity/stability) on thyroid function remained unevaluated.

Type of DM: The study grouped T1DM and T2DM patients. The prevalence and patterns of thyroid dysfunction differ significantly between these types (higher in type 1 due to autoimmunity). Separate analysis was not performed. 

Selection bias: As an outpatient-based study excluding known thyroid disease and several comorbidities, it may not represent the full spectrum of diabetic patients, particularly those with severe complications or hospitalized individuals.

Recommendations

With 30.35% of subjects exhibiting thyroid dysfunction, this study highlights its common occurrence, primarily SCH, among diabetic patients without prior thyroid diagnosis. Female gender and insulin therapy are significant risk factors. Based on these findings, we recommend the following:

Routine thyroid screening: Implement routine screening of thyroid function test (TSH, FT4) in all patients diagnosed with DM, regardless of symptoms, given the high prevalence found.

Targeted vigilance: Exercise heightened vigilance for thyroid dysfunction, particularly SCH, in female diabetic patients and those requiring insulin therapy.

SCH monitoring: Closely monitor patients with SCH for progression to overt disease and assess cardiovascular risk factors.

Future research

Future research must consider the following: (1) prospective longitudinal studies to establish causality and temporal relationships; (2) multi-center studies to enhance generalizability; (3) research incorporating HbA1c and TPO-Ab/Tg-Ab and stratifying by DM type (type 1 vs. type 2) to provide deeper mechanistic insights and refine screening strategies; (4) studies examining the impact of treating SCH on diabetes outcomes (glycemic control, complications) in this population; (5) routine screening and appropriate management of thyroid disorder in diabetic patients represent a simple yet crucial step towards optimizing their overall endocrine health and potentially improving long-term outcomes.

## Conclusions

The findings from this investigation demonstrate a considerable clinical overlap between thyroid dysfunction and DM, underscoring thyroid disorders as a frequent comorbidity in diabetic individuals. The spectrum of dysfunction is predominantly characterized by subclinical hypothyroidism, and, notably, the likelihood of its occurrence does not correlate with the length of a patient's diabetic history. A significantly elevated risk was, however, observed among female patients and those whose treatment regimen includes insulin therapy, defining these as key populations for focused assessment.

Therefore, integrating systematic thyroid function testing into the standard management protocol for all diabetes patients is strongly advocated. This proactive approach to screening ensures the early identification and subsequent management of thyroid abnormalities, which is a fundamental strategy for achieving optimal glycemic control, reducing the burden of cardiovascular complications, and decreasing overall diabetes-related morbidity. The adoption of such a practice is a vital step towards delivering holistic and effective patient care.
